# Associations between common mental disorders, cardiovascular risk factors and lifestyle in adults participating in targeted health dialogues in Sweden

**DOI:** 10.1016/j.pmedr.2026.103474

**Published:** 2026-04-16

**Authors:** Ulrika Andersson, Emelie Stenman, Anton Grundberg, Kristina Sundquist

**Affiliations:** a Center for Primary Health Care Research, Department of Clinical Sciences Malmö, Lund University, Malmö, Sweden; b University Clinic Primary Care, Skåne University Hospital, Region Skåne, Sweden

**Keywords:** Anxiety disorders, Cardiovascular risk factors, Depressive disorders, Primary healthcare, Primary prevention, Stress-related disorders

## Abstract

**Objective:**

To examine associations of common mental disorders (CMDs) with lifestyle habits and cardiovascular risk factors among participants in targeted health dialogues in Sweden.

**Methods:**

A cross-sectional study included 8903 40- and 50-year-olds between September 20, 2021, and January 10, 2024. CMDs included depressive, anxiety, and stress-related disorders. Associations between CMDs and lifestyle habits/cardiovascular risk factors were examined using logistic regression, adjusted for sex, education, marital status, and place of birth.

**Results:**

A quarter of the participants had a CMD. Those without a mental diagnosis constituted the controls. Depressive disorders were associated with physical inactivity (OR 1.43, 95% CI 1.17, 1.76), obesity (OR 1.42, 95% CI 1.16, 1.74), and tobacco use (OR 1.69, 95% CI 1.35, 2.10). Anxiety was associated with excessive alcohol use (OR 1.27, 95% CI 1.06, 1.51), unhealthy diet (OR 1.15, 95% CI 1.00, 1.33), tobacco use (OR 1.83, 95% CI 1.57, 2.14), and higher cardiovascular risk (SCORE2; OR 1.32, 95% CI 1.05, 1.64). Stress-related disorders were associated with tobacco use (OR 1.35, 95% CI 1.10, 1.64) and elevated LDL-cholesterol (OR 1.17, 95% CI 1.01, 1.37).

**Conclusions:**

Detection of CMDs in middle-aged primary care populations may offer an opportunity for early lifestyle intervention and cardiovascular risk reduction.

## Highlights

• Common mental disorders were associated with an increased cardiovascular risk.
• Adverse lifestyle behaviours were linked to common mental disorders.
• Cardiovascular risk factor profiles differed by psychiatric diagnosis.

## 1 Introduction

Common mental disorders (CMDs), including depressive, anxiety and stress-related disorders are an increasing global health challenge (Fan et al., 2025). CMDs have been associated with an elevated cardiovascular risk, potentially through pathways involving lifestyle habits and physiological dysregulation (Firth et al., 2019; Song et al., 2019). Lifestyle factors have been proposed as potential mediators in the relationship between CMD and cardiovascular disease (CVD), but empirical evidence remains mixed, and the directionality of these associations is not fully understood. Anxiety disorders have been associated with an increased risk of cigarette smoking and nicotine dependence, and physical activity with a lower risk of depression. Cardiovascular risk factors such as obesity and hypertension appear to be bidirectionally associated with CMDs through shared behavioural, inflammatory, and neuroendocrine pathways. However, further research is required to clarify the specific relationships (Firth et al., 2019).

In Sweden, CMDs are mainly managed within primary healthcare, where one-third of patients are estimated to have a diagnosis of a mental disorder, with anxiety, depression, and stress-related conditions predominating (Taloyan et al., 2023). In 2020, Targeted Health Dialogues (THD) were initiated in primary care in Region Scania to enhance cardiovascular health in the broader population, including people with or without somatic and mental diagnoses. Originally implemented in Sweden in 1985, THD has been associated with reduced cardiovascular mortality (Borjesson et al., 2025; The Swedish Association of Local Authorities and Regions, 2022). It has been suggested that THD can be a valuable tool in reducing cardiovascular risk for patients with mental disorders (Pikkemaat et al., 2022). All Scania residents should be invited to a THD at ages 40 and 50, creating a largely unselected middle-aged cohort suitable for assessment of cardiovascular risk factors. We previously observed associations between self-reported mental distress symptoms and cardiovascular risk factors among THD participants (Milos Nymberg et al., 2024), but these findings have not been confirmed in those with mental diagnoses.

This study is grounded in the Biopsychosocial Model, which proposes that health outcomes are the result of complex interactions among biological, psychological, and social factors (Engel, 1977). It has previously been applied to research on depression and diabetes and is particularly relevant in primary care research as it provides a holistic framework (Amsah et al., 2022).

The implementation of THDs in Region Scania offers an opportunity to explore how CMDs relate to modifiable cardiovascular risk factors in middle-aged individuals residing in a sociodemographically highly diverse metropolitan region in southern Sweden. The population-based data from primary care settings in a country with universal health care will make the findings of our study generalizable to many other similar countries. Thus, the present study aims to examine associations of CMDs – including depressive, anxiety, and stress-related disorders – with lifestyle behaviour and cardiovascular risk factors. In this study, these factors are treated as measurable indicators of potential mediators linked to CMDs rather than analysing their mediating effects. This is because multiple mechanisms may underly the potential associations and because cross-sectional data is not ideal for examining mediating effects. We hypothesize that individuals with CMDs exhibit a higher prevalence of unhealthy lifestyle behaviours and elevated cardiovascular risk.

## 2 Methods

### 2.1 Study design and population

This cross-sectional study involved participants the year they turned 40 or 50 years and attended a THD at a Primary Health Care Centre (PHCC) in the Scania region between September 20, 2021, and January 10, 2024. All THD participants were invited to a research project, and written consent was a prerequisite for participation. Participants received no financial or other compensation. Region Scania, Sweden's third most populous region with 1.4 million inhabitants, is ethnically diverse, with about a quarter of the population born abroad (Region Skåne, 2024a). In total, there are around 180 PHCCs (Region Skåne, 2024b). In 2021, it was decided by the County Council to continuously implement THDs at all PHCCs (public and private). The THDs are conducted by trained health professionals and preceded by clinical assessments one week earlier (fasting blood samples, blood pressure, body mass index (BMI), waist-hip-ratio) along with a questionnaire concerning background characteristics, lifestyle, family history, and symptoms. The results are presented in a “health profile”, which serves as a basis for the dialogue between the individual and the professional.

**Fig. 1:**
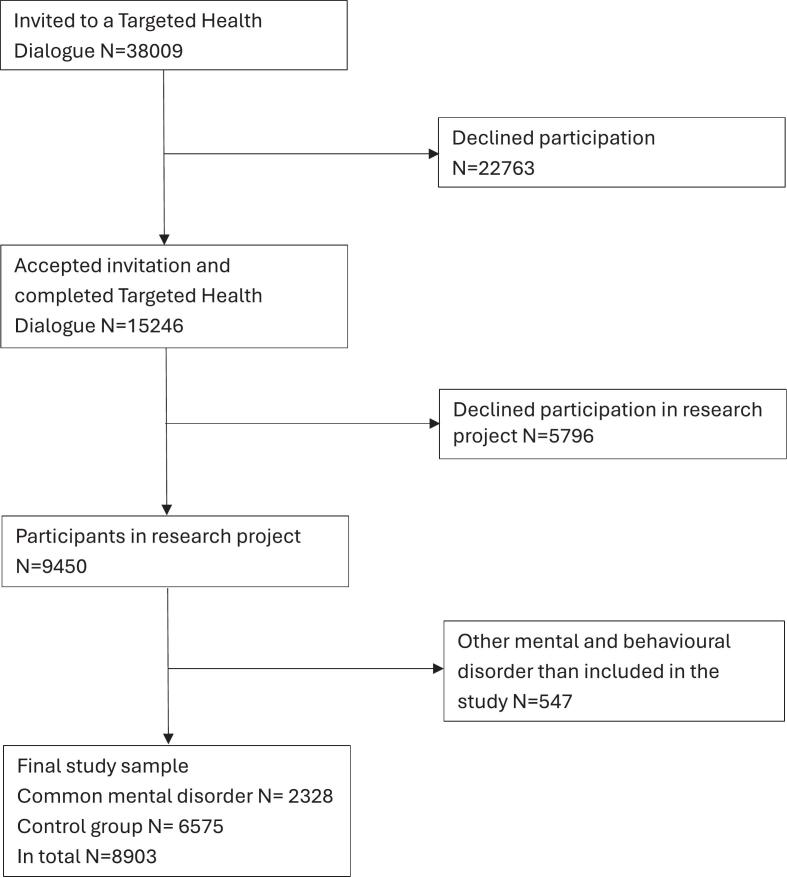
Recruitment flowchart for the Targeted Health Dialogue research project. Residents in Region Skåne were invited to at Targeted Health Dialogue at their local primary health care centre the year they turned 40 or 50 years between September 20, 2021, and January 10, 2024.

A total of 7103 40-year-olds and 2347 50-year-olds participated in a THD and consented to participation in the study (Fig. 1), corresponding to 62% of all individuals completing a THD during the study period. Of these, 547 individuals were found to have other mental diagnoses than those relevant to this study and were excluded, and 2328 individuals (24.6%) were diagnosed with a CMD in the past three years. The remaining 6575 individuals without any mental, behavioural or neurodevelopmental disorders served as the reference group.

### 2.2 Measures

CMD diagnoses were extracted from primary and secondary healthcare in Region Scania. The diagnoses were classified according to the International Classification of Diseases 10th revision, adapted for Swedish conditions (ICD-10-SE). Included in the term CMDs in this study were depressive disorders (F32 and F33), anxiety disorders (F41) and stress-related disorders (F43), as they are the most prevalent diagnoses in Swedish primary healthcare and account for a substantial morbidity burden. Depressive and anxiety disorders are among the leading contributors to the non-fatal disease burden globally (Sundquist et al., 2017; Vos et al., 2020). We excluded individuals with post-traumatic stress disorder (F43.1) as this condition was considered to differ in clinical presentation and pathogenesis from other stress-related disorders (Ressler et al., 2022). A CMD diagnosis during the past three years was considered relevant, assuming potential health and lifestyle effects despite unknown current status.

Individuals with no recent history of mental disorders, defined as no ICD-code F01-99 (mental, behavioural and neurodevelopmental disorders) in the last three years, were used as a reference group. If a participant had more than one of the included diagnoses registered in the past three years, the most recent diagnosis was used.

Information about lifestyle habits and cardiovascular risk factors was derived from the self-reported questionnaire as well as from clinical measures and laboratory tests. Parts of the questionnaire have been validated previously (Johansson et al., 2023; Persson et al., 1998), but the psychometric properties were not validated in the current study sample. The included variables were selected based on available data from the THDs and their established relevance to cardiovascular health. Low-density lipoprotein (LDL, mmol/L) and plasma glucose (mmol/L) were measured by a fasting blood test one week before the THD, while BMI (kg/m^2^) and blood pressure (mmHg) were measured at the PHCC. Blood pressure was measured in a sitting position in both arms at least twice with one minute in between, and the mean value was registered. Physical activity was assessed with the question “*How physically active are you in your leisure time?”* with response options: 1) sedentary leisure time, 2) moderate exercise, 3) strenuous exercise, and 4) hard exercise. Participants who chose alternatives 2 or 3 were asked to respond to follow-up questions regarding the mode of transport to work and leisure time physical activities. Physical activity was reported as minutes per week and season and multiplied with specific energy factors for different activities (range 0-10 points). Expending less than 2000 kcal per week during leisure time was considered insufficient physical activity (Persson et al., 1998). Dietary habits were evaluated by 28 questions adapted from a validated THD questionnaire (Lingfors et al., 1994). The diet score within the health profile was derived from a fat and fibre index. If participants indicated consuming “sweets, chocolate, or sugar-sweetened beverages” or “cakes or cookies” twice of more per day, or both categories once daily, they were shifted to a higher risk level of the health profile. Unhealthy eating habits were defined as not being in the healthiest diet category of the health profile. Excessive alcohol intake was defined as consuming five or more standard glasses of alcohol per week for women, and seven or more for men. Drinking four or more glasses per occasion for women, and five or more for men, at least once a month, was also classified as excessive alcohol intake. The cut points were adapted from the Swedish recommendations at that time (Socialstyrelsen, 2018, no longer available online). Tobacco use was defined as using any tobacco product regularly, including smoking cigarettes or e-cigarettes, using “snus” (oral tobacco product) or waterpipe. The cardiovascular risk factors were dichotomised in the regression analyses. Blood pressure was defined as high if ≥140/90 mmHg, consistent with the diagnostic criteria of hypertension (McEvoy et al., 2024). Obesity was defined as BMI ≥30 kg/m^2^, in alignment with the WHO definition (World Health Organization, 2025). The cut-off value for fasting glucose level was set to ≥6.1 mmol/L, in line with the WHO definition of impaired fasting glucose (World Health Organization and International Diabetes Federation, 2006). As the cut-off value for an optimal level of LDL varies according to comorbidities and total cardiovascular risk, we used the mean value in the control group as the non-sex-specific cut-off value (3.27 mmol/L). Cardiovascular risk was assessed using the algorithm SCORE2, adopted for Sweden (Hageman et al., 2021). Given the low estimated 10-year risk of CVD morbidity and/or mortality in these age groups, results were dichotomised by merging the medium- and high-risk groups to allow adequate subgroup sizes for meaningful analyses. Preventive treatment may be indicated among individuals with increased risk, depending on the overall cardiovascular profile and clinical judgement.

### 2.3 Statistical analysis

Differences between the reference group and each CMD group were analysed using pairwise comparisons: Pearson's chi^2^-tests for all categorical variables (except SCORE2 for which Fisher's exact tests were used as they contained expected values <5 in more than 20% of the cells), and Student's *t*-tests for the continuous and normally distributed LDL cholesterol (Table 1). Thereafter, logistic regression models were used to examine associations between CMDs treated as independent variables, and lifestyle and cardiovascular risk factors treated as outcomes. Results are presented as odds ratios (ORs) with a 95% confidence interval (CI). The models were adjusted for sex, education level, marital status and place of birth, as these variables were considered to represent possible confounders based on their potential influence on both the exposures and outcomes (Table 2, Table 3). The chosen level of statistical significance was 0.05. All statistical analyses were performed with R, version 4.4.2.

**Table 1:** Lifestyle behaviours and cardiovascular risk factors among 40- and 50-year-olds attending targeted health dialogues in Region Scania, Sweden (2021–2024), stratified by common mental disorders.

Characteristic	No CMD, n (%)	Any included CMD, n (%)	Depressive disorders⁎, n (%)	Anxiety disorders⁎, n (%)	Stress-related disorders⁎, n (%)
Physical activity					
Sufficient	2189 (33.3)	709 (30.5)	136 (26.2)	367 (31.3)	255 (31.8)
Insufficient	4208 (64.0)	1552 (66.7)	368 (70.9)	772 (65.8)	521 (64.9)
Missing	178 (2.7)	67 (2.9)	15 (2.9)	34 (2.9)	27 (3.4)
*p-value*⁎⁎		*0.01*	*<0.01*	*0.20*	*0.45*

Eating habits					
Healthy	2109 (32.1)	659 (28.3)	144 (27.7)	335 (28.6)	230 (28.6)
Unhealthy	3448 (52.4)	1278 (54.9)	282 (54.3)	650 (55.4)	435 (54.2)
Missing	1018 (15.5)	391 (16.8)	93 (17.9)	188 (16.0)	138 (17.2)
*p-value*⁎⁎		*<0.01*	*0.09*	*0.02*	*0.09*

Excessive alcohol consumption					
No	5441 (82.8)	1916 (82.3)	435 (83.8)	935 (79.7)	682 (84.9)
Yes	924 (14.1)	311 (13.4)	61 (11.8)	187 (15.9)	85 (10.6)
Missing	210 (3.2)	101 (4.3)	23 (4.4)	51 (4.3)	36 (4.5)
*p-value*⁎⁎		*0.55*	*0.18*	*0.06*	*0.01*

Tobacco use					
No	5426 (82.5)	1787 (76.8)	383 (73.8)	866 (73.8)	648 (80.7)
Yes	1080 (16.4)	522 (22.4)	133 (25.6)	295 (25.1)	149 (18.6)
Missing	69 (1.0)	19 (0.8)	3 (0.6)	12 (1.0)	6 (0.7)
*p-value*⁎⁎		*<0.01*	*<0.01*	*<0.01*	*0.14*

BMI categorical					
< 25	2852 (43.4)	1011 (43.4)	222 (42.8)	511 (43.6)	349 (43.5)
25–29.9	2401 (36.5)	777 (33.4)	157 (30.3)	389 (33.2)	281 (35.0)
≥ 30	1301 (19.8)	528 (22.7)	139 (26.8)	266 (22.7)	169 (21.0)
Missing	21 (0.3)	12 (0.5)	1 (0.2)	7 (0.6)	4 (0.5)
*p-value*⁎⁎		*<0.01*	*<0.01*	*0.03*	*0.60*

Blood pressure categorical					
< 140/90	5070 (77.1)	1826 (78.4)	411 (79.2)	907 (77.3)	646 (80.4)
≥ 140/90	1486 (22.6)	493 (21.2)	106 (20.4)	261 (22.3)	154 (19.2)
Missing	19 (0.3)	9 (0.4)	2 (0.4)	5 (0.4)	3 (0.4)
*p-value*⁎⁎		*0.17*	*0.26*	*0.81*	*0.03*
LDL cholesterol, mean (SD)	3.27 (0.91)	3.25 (0.94)	3.36 (1.01)	3.22 (0.91)	3.23 (0.93)
*p-value*⁎⁎		*0.34*	*0.06*	*0.06*	*0.24*

F-plasma glucose					
≤6 mmol/l	5846 (88.9)	2098 (90.1)	464 (89.4)	1053 (89.8)	732 (91.2)
6.1–6.9 mmol/l	528 (8.0)	167 (7.2)	34 (6.6)	83 (7.1)	56 (7.0)
≥7 mmol/l	141 (2.1)	43 (1.8)	16 (3.1)	24 (2.0)	12 (1.5)
Missing	60 (0.9)	20 (0.9)	5 (1.0)	13 (1.1)	3 (0.4)
*p-value*⁎⁎		*0.27*	*0.20*	*0.53*	*0.24*

SCORE2					
Low risk	5806 (88.3)	2073 (89.0)	453 (87.3)	1031 (87.9)	730 (90.9)
Medium risk	699 (10.6)	217 (9.3)	56 (10.8)	120 (10.2)	61 (7.6)
High risk	18 (0.3)	11 (0.5)	3 (0.6)	6 (0.5)	3 (0.4)
Missing	52 (0.8)	27 (1.2)	7 (1.3)	16 (1.4)	9 (1.1)
*p-value*⁎⁎		*0.08*	*0.35*	*0.35*	*0.02*

CMD, common mental disorder; BMI, body mass index; LDL, low density lipoprotein; SD, standard deviation.

⁎ Depressive disorders included ICD-10 codes F32 and F33; Anxiety disorders F41; Stress-related disorders F43.

⁎⁎ Pairwise comparisons with the reference group (no CMD). LDL cholesterol: Student's t-tests. SCORE2: Fisher's exact tests. All other variables: Pearson's chi^2^-tests.

**Table 2:** Associations between common mental disorders and lifestyle behaviours among 40- and 50-year-olds attending targeted health dialogues in Region Scania, Sweden (2021–2024).

	Insufficient physical activity⁎		Unhealthy eating habits⁎		Excessive alcohol consumption⁎		Tobacco use⁎	
	OR (95% CI)		OR (95% CI)		OR (95% CI)		OR (95% CI)	
	Unadjusted	Adjusted⁎⁎	Unadjusted	Adjusted⁎⁎	Unadjusted	Adjusted⁎⁎	Unadjusted	Adjusted⁎⁎
Any included CMD	1.14 (1.03, 1.26)	1.19 (1.07, 1.32)	1.19 (1.06, 1.32)	1.17 (1.05, 1.31)	0.96 (0.83, 1.10)	1.00 (0.86, 1.15)	1.47 (1.30, 1.65)	1.76 (1.55, 2.00)
Depression (F32 + F33)	1.39 (1.14, 1.70)	1.43 (1.17, 1.76)	1.16 (0.94, 1.42)	1.13 (0.92, 1.39)	0.83 (0.62, 1.08)	0.79 (0.59, 1.04)	1.61 (1.31, 1.98)	1.69 (1.35, 2.10)
Anxiety (F41)	1.07 (0.93, 1.22)	1.09 (0.95, 1.24)	1.16 (1.01, 1.33)	1.15 (1.00, 1.33)	1.23 (1.03, 1.45)	1.27 (1.06, 1.51)	1.65 (1.43, 1.91)	1.83 (1.57, 2.14)
Stress (F43)	1.03 (0.88, 1.21)	1.07 (0.92, 1.26)	1.12 (0.95, 1.32)	1.10 (0.93, 1.30)	0.72 (0.57, 0.91)	0.79 (0.62, 1.01)	1.04 (0.86, 1.25)	1.35 (1.10, 1.64)

OR, odds ratio; CI, confidence interval; CMD, common mental disorder.

⁎ Insufficient physical activity was defined as expending less than 2000 kcal per week during leisure time; unhealthy eating habits as not being in the healthiest diet category of the health profile; excessive alcohol consumption as weekly (≥5/≥7 drinks) or monthly binge drinking (≥4/≥5 drinks) for women/men; and tobacco use as using any tobacco product regularly.

⁎⁎ Adjusted for sex, level of education, marital status and place of birth.

**Table 3:** Associations between common mental disorders and cardiovascular risk factors among 40- and 50-year-olds attending targeted health dialogues in Region Scania, Sweden (2021–2024).

	Obesity (BMI ≥30 kg/m^2^)		High blood pressure (>140/90 mmHg)		High LDL cholesterol (>3.27 mmol/L)		High f-plasma glucose (≥6.1 mmol/L)		High cardiovascular risk (SCORE2, ≥2,5% for 40-year-olds, ≥5% for 50-year-olds)	
	OR (95% CI)		OR (95% CI)		OR (95% CI)		OR (95% CI)		OR (95% CI)	
	Unadjusted	Adjusted⁎	Unadjusted	Adjusted⁎	Unadjusted	Adjusted⁎	Unadjusted	Adjusted⁎	Unadjusted	Adjusted⁎
Any included CMD	1.19 (1.06, 1.34)	1.20 (1.06, 1.35)	0.92 (0.82, 1.03)	1.00 (0.89, 1.13)	0.99 (0.90, 1.09)	1.14 (1.03, 1.26)	0.86 (0.60, 1.20)	0.93 (0.64, 1.33)	0.89 (0.76, 1.04)	1.34 (1.12, 1.59)
Depression (F32 + F33)	1.45 (1.18, 1.76)	1.42 (1.16, 1.74)	0.89 (0.71, 1.11)	0.90 (0.72, 1.12)	1.09 (0.91, 1.30)	1.16 (0.96, 1.39)	1.56 (0.89, 2.54)	1.56 (0.87, 2.60)	1.09 (0.82, 1.43)	1.34 (0.97, 1.82)
Anxiety (F41)	1.16 (1.00, 1.34)	1.13 (0.97, 1.31)	1.00 (0.86, 1.16)	1.04 (0.89, 1.21)	0.96 (0.85, 1.09)	1.03 (0.91, 1.17)	0.99 (0.63, 1.50)	1.01 (0.63, 1.55)	1.02 (0.83, 1.24)	1.32 (1.05, 1.64)
Stress (F43)	1.04 (0.86, 1.24)	1.09 (0.91, 1.31)	0.82 (0.68, 0.98)	0.95 (0.78, 1.14)	0.97 (0.83, 1.12)	1.17 (1.01, 1.37)	0.70 (0.36, 1.20)	0.80 (0.41, 1.42)	0.72 (0.54, 0.92)	1.27 (0.94, 1.70)

OR, odds ratio; CI, confidence interval; CMD, common mental disorder; BMI, body mass index; LDL, low density lipoprotein.

⁎ Adjusted for sex, level of education, marital status and place of birth.

## 3 Results

Participants with CMDs were more often women (66.8% vs. 52.2%), had higher levels of education (>12 years: 67.6% vs. 64.7%), were more likely to be Swedish-born (74.9% vs. 68.2%), and were less likely to be married or cohabiting (71.2% vs. 84.6%) compared to the reference group. Differences in education level were observed between the CMD groups, with the overall higher education level among participants with CMDs primarily driven by those with stress-related disorders, who had a significantly higher proportion of individuals with >12 years of education compared to the reference group (72.5%, *p* < 0.01). The age distribution was generally similar, although individuals with anxiety disorders were somewhat more often aged 40 years (80.2%) compared to the reference group (74.6%). The distribution of lifestyle behaviours and cardiovascular risk factors is presented in Table 1 (sociodemographic characteristics are not shown in tables). Anxiety disorders were the most common mental disorder (*n* = 1173, 12.4%). Stress-related disorders were the second most common (*n* = 807, 8.5%), and depressive disorders were the least common (*n* = 519, 5.5%).

The logistic regression analyses for health behaviours (Table 2) showed a significant association between depressive disorders and insufficient physical activity (unadjusted OR 1.39, 95% CI 1.14, 1.70; adjusted OR 1.43, 95% CI 1.17, 1.76). Anxiety was significantly associated with excessive alcohol consumption (unadjusted OR 1.23, 95% CI 1.03, 1.45; adjusted OR 1.27, 95% CI 1.06, 1.51), unhealthy eating (unadjusted OR 1.16, 95% CI 1.01, 1.33; adjusted OR 1.15, 95% CI 1.00, 1.33), and tobacco use (unadjusted OR 1.65, 95% CI 1.43, 1.91; adjusted OR 1.83, 95% CI 1.57, 2.14). Depressive disorders (unadjusted OR 1.61, 95% CI 1.31, 1.98; adjusted OR 1.69, 95% CI 1.35, 2.10) and stress-related disorders (unadjusted OR 1.04, 95% CI 0.86, 1.25; adjusted OR 1.35, 95% CI 1.10, 1.64) were also significantly associated with tobacco use. Regarding cardiovascular risk factors (Table 3), individuals with depressive disorders were obese to a higher extent (unadjusted OR 1.45, 95% CI 1.18, 1.76; adjusted OR 1.42, 95% CI 1.16, 1.74). There was no significant association between high blood pressure or high fasting glucose levels and any of the mental diagnoses after adjustment for potential confounders. Stress-related disorders were associated with high LDL cholesterol (unadjusted OR 0.97, 95% CI 0.83, 1.12; adjusted OR 1.17, 95% CI 1.01, 1.37). Anxiety was significantly associated with high SCORE2 (unadjusted OR 1.02, 95% CI 0.83, 1.24; adjusted OR 1.32, 95% CI 1.05, 1.64).

## 4 Discussion

Among the 40- and 50-year-old participants, CMDs were more prevalent in women, those with higher education, Swedish-born, and/or those who were not married or cohabiting. Associations between CMDs and lifestyle and cardiovascular risk factors varied across diagnoses: depressive disorders were associated with higher odds of insufficient physical activity and obesity, anxiety with higher odds of unhealthy eating and excessive alcohol use, and all CMDs with higher odds of tobacco use. Stress-related disorders were associated with higher odds of high LDL cholesterol.

Nearly a quarter of the study participants had been diagnosed with a CMD within three years preceding the THD, which may appear high. A study from 2017 reported a one-year prevalence of 4.9% for anxiety, 3.8% for depression, and 2.7% for stress-related disorders in the Stockholm region (Forslund et al., 2020). Another Swedish study estimated the prevalence of depression in primary healthcare to be 12.4%, and anxiety and stress-related disorders at 9.9% and 9.2%, respectively (Sundquist et al., 2017). Thus, the magnitude of CMDs in our study population appears to be comparable to the general population, although the distribution differed somewhat, with depressive disorders being the least common diagnosis. The differences may reflect regional differences in diagnostic practices and healthcare-seeking behaviours, but also the recruitment context of THDs, which includes specific age groups and could attract selected groups of individuals. As in previous studies, women were overrepresented (Forslund et al., 2020; Sundquist et al., 2017). Previous research suggests that individuals born outside of Europe report more symptoms of anxiety and depression but have fewer registered diagnoses in the Swedish healthcare system (Centrum för epidemiologi och samhällsmedicin Region Stockholm, 2023), which aligns with our results regarding participant characteristics.

Although the cross-sectional design of this study precludes causal inference, the patterns are consistent with previous literature on reciprocal relationships between CMDs and lifestyle and cardiovascular risk factors (D'Aquino et al., 2024; Gerardo et al., 2025). Some individuals may adopt healthier behaviours as part of their treatment, while unhealthy lifestyles may contribute to the onset of a CMD. For example, smoking has been implicated as a potential risk factor for major depression, whereas physical activity is associated with reduced risk, which is consistent with the present study as depressive disorders were associated with higher odds of tobacco use and insufficient physical activity. Exercise has demonstrated positive effects in treating CMDs (Firth et al., 2020). The role of diet is less clear, though some evidence supports beneficial effects of dietary interventions on CMDs (Firth et al., 2020). Depressive disorders were also significantly associated with higher odds of obesity. Previous literature indicates that the relationship between depression and obesity is multifactorial and bi-directional: obesity has been associated with depression through mechanisms such as inflammation, hormonal imbalances, and social stigma, while depression may in turn be linked to obesity through inactivity and overeating (Gerardo et al., 2025). Weight gain is also a common side effect of many antidepressant medications (Fulton et al., 2022). However, in the present study causality cannot be determined, underscoring the need for further research.

Tobacco use in our study included “snus”, which constituted a substantial part of the tobacco use reported by the participants (Stenman et al., 2024). Although “snus” is a form of tobacco, its association with increased cardiovascular risk appears to be weaker compared to smoking (Clarke et al., 2019; Titova et al., 2021). Nevertheless, it should not be recommended as a substitute for smoking. In SCORE2, smoking is included, but no other tobacco products. All included conditions showed indications of an association with higher cardiovascular risk over time according to the SCORE2 classification, and the odds were significantly higher for anxiety. A Swedish primary care study reported that patients with alcohol use disorder exhibited high odds of comorbid mental disorder (Taloyan et al., 2023). In our dataset, anxiety was positively associated with higher odds of alcohol use, whereas the relationship between alcohol and depressive and stress-related disorders appeared to be inverse. This illustrates the importance of differentiating between CMDs, as their associated risk factors and lifestyle habits may vary. Previous studies have reported overlapping but distinct behavioural risk profiles (D'Aquino et al., 2024; Firth et al., 2019), suggesting that intervention strategies may need to be tailored by disorder type. The association between anxiety and excessive alcohol consumption is well documented, although causality is less clear. Previous literature indicates that alcohol may be used as self-medication to alleviate anxiety, yet over time it may also contribute to worsening anxiety levels (D'Aquino et al., 2024). Notably, fewer individuals with stress-related disorders reported excessive alcohol consumption compared to the rest of the study population, although not significantly so when adjusting for sex, level of education, marital status, and place of birth, which aligns with findings from previous research, indicating less unhealthy behaviours in this group (Grossi et al., 2022). Overall, our findings reinforce the interconnectedness of mental health, lifestyle, and cardiovascular risk factors. While the patterns observed are broadly consistent with previous literature, the distinct associations across CMDs suggest that associative mechanisms and behavioural responses may differ by diagnosis. Future longitudinal studies are warranted to clarify the temporal and causal pathways linking CMDs with lifestyle and cardiovascular health.

One limitation of the study is its cross-sectional design, which makes inferences regarding causality impossible. Participants were diagnosed up to three years prior to their THD, and the status of remission and symptoms at the time of the THD was unknown, limiting conclusions about the temporal relationship between lifestyle and cardiovascular risk factors and CMDs. An additional limitation related to cardiovascular risk factors is that we lack information on medication use. Logistic regression was used to analyse associations. Since both CMDs and lifestyle behaviour and cardiovascular risk factors are common in the sample, ORs may be inflated. However, given that the estimated odds ratios in this study were generally quite low (<2), the potential inflation is likely negligible. The study population's age was defined by political decisions, limiting generalizability to other age groups. Recruitment through THDs may attract more health-conscious individuals than the general population. One previous study indicated, for example, that male, low-educated, and foreign-born individuals were slightly under-represented in THD participation (Stenman et al., 2024). Lifestyle variables were derived from self-reported data, which may introduce reporting bias. As participants were well informed about the THD's objectives and likely to be motivated to improve their health, their responses are assumed to be largely accurate and reliable. Psychometric properties were not available for some of the self-reported variables. However, because the same questions were administered to both groups and we adjusted for several confounders, it was possible to reduce the impact of this limitation. A key strength of this study is the relatively unselected study population, as all residents are invited to participate in a THD at ages 40 and 50 without exclusion criteria. This enhances the generalisability of the findings. An additional strength is the study setting in Region Scania, which includes both rural and metropolitan areas and a culturally diverse population, further supporting the external validity of the results.

## 5 Conclusion

Our findings demonstrate that CMDs in 40- and 50-year-olds are associated with higher odds of unhealthy lifestyle behaviours. These findings may be relevant to public health research, clinical practice and policy, underscoring the need for further studies on causal relationships and diagnosis-specific risk profiles. Given the limited resources in primary care, preventive and therapeutic interventions may be prioritised toward groups with the most elevated cardiovascular risk profiles. Lifestyle interventions may need to be adapted to specific CMDs, focusing on physical activity and weight management in depressive disorders, reducing alcohol use in anxiety, and tobacco cessation across all CMDs. Differentiating between subtypes of mental disorders may enhance the effectiveness of personalised preventive strategies and public health interventions. Integrated strategies in primary care, such as targeted health dialogues, could potentially help identify and address multiple risk factors, although further research is needed to establish their effectiveness in relation to the costs.

## CRediT authorship contribution statement

**Ulrika Andersson:** Writing – review & editing, Writing – original draft, Methodology, Data curation, Conceptualization. **Emelie Stenman:** Writing – review & editing, Project administration, Methodology, Formal analysis, Data curation, Conceptualization. **Anton Grundberg:** Writing – review & editing, Formal analysis, Conceptualization. **Kristina Sundquist:** Writing – review & editing, Supervision, Project administration, Funding acquisition, Conceptualization.

## Ethical approval

Ethical approval for the research project was obtained from the Swedish Ethical Review Authority (registration number 2020-02689 with amendments). The study was registered at ClinicalTrials.gov, identifier: NCT04912739.

## Funding sources

This work was supported by funding to Kristina Sundquist from the Swedish Heart Lung Foundation (20210553, and 2024128926), the Swedish Research Council (2021-06456) and from the Swedish state under the agreement between the Swedish government and the county councils, the ALF-agreement. The authors declare that they have no known competing financial interests or personal relationships that could have appeared to influence the work reported in this paper.

## Declaration of competing interest

The authors declare that they have no known competing financial interests or personal relationships that could have appeared to influence the work reported in this paper.

## Data Availability

Data will be made available on request.
